# Microstructure and hydrogen storage properties of the Mg_2−x_Y_x_Ni_0.9_Co_0.1_ (x = 0, 0.2, 0.3, and 0.4) alloys

**DOI:** 10.1038/s41598-024-51602-w

**Published:** 2024-01-09

**Authors:** Defa Li, Feng Huang, Bingzhi Ren, Shujie Wang, Wei Zhang, Liming Zhu

**Affiliations:** 1 School of Mechanical and Vehicle Engineering, West Anhui University, Luan, 237012 China; 2 Hubei Key Laboratory of Advanced Technology for Automotive Components, Wuhan University of Technology, Wuhan, 430070 China; 3 School of Metallurgy and Materials Engineering, Chongqing University of Science and Technology, Chongqing, 401331 China; 4 The 13th Research Institute, CETC, Shijiazhuang, 050051 China

**Keywords:** Energy science and technology, Materials science

## Abstract

Rare earth elements have excellent catalytic effects on improving hydrogen storage properties of the Mg_2_Ni-based alloys. This study used a small amount of Y to substitute Mg partially in Mg_2_Ni_0.9_Co_0.1_ and characterized and discussed the effects of Y on the solidification and de-/hydrogenation behaviors. The Mg_2−x_Y_x_Ni_0.9_Co_0.1_ (x = 0, 0.2, 0.3, and 0.4) hydrogen storage alloys were prepared using a metallurgy method. The phase composition of the alloys was studied using X-ray diffraction (XRD). Additionally, their microstructure and chemical composition were studied using scanning electron microscopy and energy-dispersive X-ray spectroscopy, respectively. The hydrogen absorption and desorption properties of the alloys were studied using pressure-composition isotherms and differential scanning calorimetric (DSC) measurements. The structure of the as-cast Mg_2_Ni_0.9_Co_0.1_ alloy was composed of the peritectic Mg_2_Ni, eutectic Mg–Mg_2_Ni, and a small amount of pre-precipitated Mg–Ni–Co ternary phases, and was converted into the Mg_2_NiH_4_, Mg_2_Ni_0.9_Co_0.1_H_4_, and MgH_2_ phases after hydrogen absorption. Furthermore, the XRD patterns of the alloys showed the MgYNi_4_ phase and a trace amount of the Y_2_O_3_ phase along with the Mg and Mg_2_Ni phases after the addition of Y. After hydrogen absorption, the phase of the alloys was composed of the Mg_2_NiH_4_, MgH_2_, MgYNi_4_, YH_3_, Y_2_O_3_, and Mg_2_NiH_0.3_ phases. With the increase of Y addition, the area ratios of the peritectic Mg_2_Ni matrix phase in the Mg_2−x_Y_x_Ni_0.9_Co_0.1_ (x = 0, 0.2, 0.3, and 0.4) alloys gradually decreased until they disappeared. However, the eutectic structure gradually increased, and the microstructures of the alloys were obviously refined. The addition of Y improves the activation performance of the alloys. The alloy only needed one cycle of de-/hydrogenation to complete the activation for x = 0.4. The DSC curves showed that the initial dehydrogenation temperatures of Mg_2_Ni_0.9_Co_0.1_ and Mg_1.8_Y_0.2_Ni_0.9_Co_0.1_ were 200 and 156 °C, respectively. The desorption activation energies of the hydrides of the Mg_2_Ni_0.9_Co_0.1_ and Mg_1.8_Y_0.2_Ni_0.9_Co_0.1_ alloys calculated using the Kissinger method were 94.7 and 56.5 kJ/mol, respectively. Moreover, the addition of Y reduced the initial desorption temperature of the alloys and improved their kinetic properties.

## Introduction

Hydrogen energy has been widely studied and is considered a green energy expected to replace petroleum because of its advantages, such as clean energy, abundant reserves, and good combustion performance^1–3^. The current research on hydrogen is mainly focused on its production, storage, transportation, and application. Although the preparation and application technology of hydrogen is relatively mature, hydrogen storage is a major problem that needs to be solved. Hydrogen storage technology is the key to widely applying hydrogen as a fuel^4^.

A_2_B type magnesium series hydrogen storage alloy, Mg_2_Ni, is regarded as one of the most promising hydrogen storage materials in the twenty-first century since it has a high hydrogen storage capacity, low price, and abundant resources. However, its disadvantages, such as slow kinetics, over-stable hydride, and high hydrogen desorbing temperature, hinder its use in practical applications^5–7^. Consequently, extensive research, including alloying^8–10^, doping of catalysts^11,12^, and fabrication of composites^13–15^, has been done to overcome this obstacle and enable it to meet the requirements of the U.S. Department of Energy for hydrogen storage materials. Many methods showed significant improvement in the hydrogen storage performance of the Mg_2_Ni type alloys by substituting Ni or Mg with transition or rare earth elements, respectively, especially its thermodynamic properties could be adjusted to enable it to meet the requirements of practical applications^16^. Song et al. believed that partially substituting Mg with Nd could improve the activation property of the Mg_2_Ni alloy^17^. Consequently, it was reported for the first time that the hydrogen absorption capacity of Mg_1.9_Nd_0.1_Ni reached 2.86 wt%, which was higher than that of Mg_2_Ni since the multiphase structure formed by substituting Mg with Nd increased the phase boundary area and provided a favorable path for the diffusion of hydrogen atoms. Kalinichenka et al. showed that the Mg–Ni–Y system is highly suitable for reversible hydrogen storage^18^. The Mg_80_Ni_10_Y_10_ and Mg_90_Ni_5_Y_5_ alloys have a high hydrogen absorption rate under the hydrogen pressure of 20 bar, even at 250 °C. According to Li et al. and Zhang et al., the addition of Y improved the hydrogen absorption and desorption thermodynamics of the MgNi-based alloys^19,20^. Xie et al. used the hydrogen plasma metal reaction (HPMR) method to successfully prepare Mg_2_Ni_1−x_Co_x_ (x = 0, 0.05, and 0.1) alloys and found that adding Co improved their hydrogen absorption kinetic properties significantly^21^.

Therefore, the literature review shows that the partial substitution of the A and B-side elements with Y and Co, respectively, contribute to improving the hydrogen storage performance of the Mg_2_Ni alloy^22–25^. However, few reports exist on the simultaneous addition of Y and Co. Thus, this study used the rare earth element Y and the transition element Co to replace Mg partially and Ni on the basis of Mg_2_Ni alloy, respectively, to realize the dual regulation of de-/hydrogenation kinetics and thermodynamic properties of the Mg_2_Ni alloy.

## Experimental procedure

### Preparation of the Mg_2−x_Y_x_Ni_0.9_Co_0.1_ samples

Commercially pure Mg (99.9% purity), Mg–Ni intermediate alloy (70 wt% Ni content, 99.9% purity), Mg–Y intermediate alloy (30 wt% Y content, 99.9% purity), and pure Co (99.9% purity) were used as raw materials to prepare Mg_2−X_Y_x_Ni_0.9_Co_0.1_ (x = 0, 0.2, 0.3, and 0.4) alloy ingots in a graphite crucible in an electric resistance furnace, under the protection of a mixed flow of SF_6_ and CO_2_. Intermittent mechanical agitation was conducted during smelting to prevent density segregation of the alloy melt. Moreover, additional Mg (2 wt%) was added to compensate for its inherent evaporation loss. Subsequently, the desired ingot could be obtained as the melt was cooled to room temperature in a furnace. The weight of each ingot was approximately 80 g.

Furthermore, the de-/hydrogenation measurement samples were taken from the center of each ingot. Prior to tests, the samples were mechanically broken, followed by ball-milling in a high-energy ball mill (HEBM) at a rotating speed of 240 rpm for about 60 min. The ball-to-material ratio during ball milling was 30:1, and high-purity argon (99.999% purity) was inserted to prevent oxidation. Additionally, intermittent ball milling was adopted to avoid the adhesion of components and excessively high temperature caused by long-duration ball milling. In intermittent ball milling, the rotation is stopped after 20 min for 15 min, then reversed until 60 min of ball milling is completed. Subsequently, about 0.5 g of 200-mesh powder was screened for the hydrogen storage performance test.

### Characterization and de-/hydrogenation measurements

The phases in the as-cast ingots and hydrogenated powders were identified using X-ray diffraction (XRD) with Cu Kα radiation for continuous scanning at a rate of 2°/min in the 2θ range of 10–85°. The microstructures were characterized using scanning electron microscopy (SEM), and the corresponding chemical compositions were analyzed using energy-dispersive X-ray spectroscopy (EDS). Meanwhile, Image-Pro Plus (IPP) was used for counting the phase area ratios in the SEM images.

After activation, the isothermal de-/hydrogenation performance of the ball-milled alloys was measured using a precise volumetric Sieverts-type apparatus at 260, 280, and 300 °C, with hydrogen pressure of 2.5 and 0.1 MPa for hydrogen absorption and desorption, respectively. The differential scanning calorimetric (DSC) measurements were conducted from room temperature to 450 °C at the heating rates of 5, 10, and 15 °C/min under 50 mL/min argon gas flow to characterize the phase transformation behaviors of the hydrides.

## Results and discussion

### Phase compositions of the as-cast alloys

**Figure 1:**
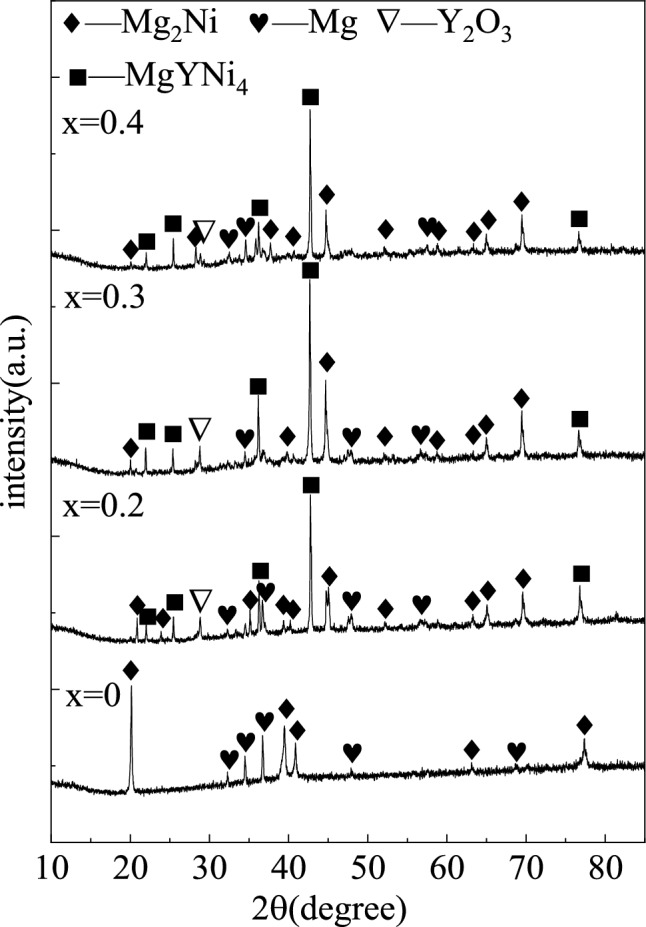
XRD patterns of the as-cast Mg_2−x_Y_x_Ni_0.9_Co_0.1_ (x = 0, 0.2, 0.3, and 0.4) alloys.

**Figure 2:**
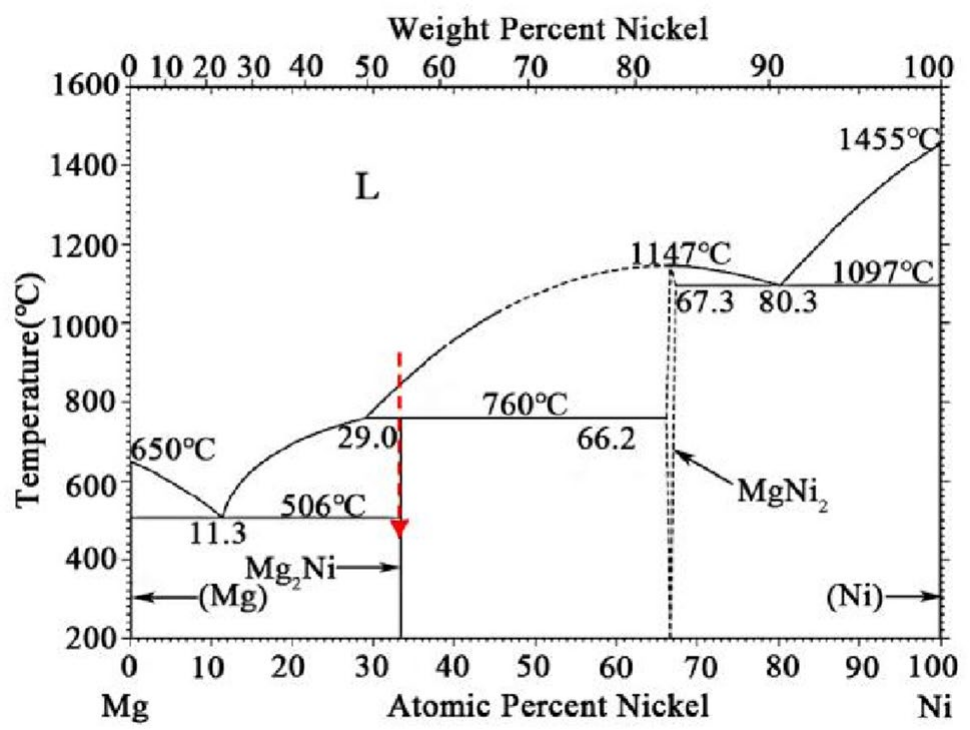
The Mg–Ni binary alloy phase diagram.

The XRD patterns of the as-cast Mg_2−x_Y_x_Ni_0.9_Co_0.1_ (x = 0, 0.2, 0.3, and 0.4) alloys are shown in Fig. 1. The diffraction peaks of the tested alloys were pointy, indicating their crystallization characteristics. Mg_2_Ni_0.9_Co_0.1_ was only composed of the Mg and Mg_2_Ni phases. As shown in the Mg–Ni binary alloy diagram in Fig. 2, the solidification path of Mg_2_Ni under equilibrium solidification conditions should be: L → L + MgNi_2_ → L + Mg_2_Ni → Mg + Mg_2_Ni. The absence of the MgNi_2_ peaks in the Mg_2_Ni_0.9_Co_0.1_ diffraction profile indicated that all the primary MgNi_2_ was transformed into Mg_2_Ni though a peritectic reaction with liquid at 760 °C or the residual MgNi_2_ was rare and diffused. Thus, the alloy was near-equilibrium solidified during furnace cooling in this study.

MgYNi_4_ and Y_2_O_3_ diffraction peaks were obviously present in the XRD patterns of Mg_2−x_Y_x_Ni_0.9_Co_0.1_ (x = 0.2, 0.3, and 0.4) along with Mg and Mg_2_Ni diffraction peaks when Y was added to substitute Mg partially. MgYNi_4_ belonged to Laves phase and had a C15 structure, which agreed well with Reference^26^. According to Reference^26^, the added Y was first dissolved in primary MgNi_2_ to replace Mg, and then MgNi_2_ reached the composition and transformed into MgYNi_4_ when the Y content increased, and the Mg content decreased in MgNi_2_. Meanwhile, it can be inferred from the high diffraction intensity shown in Fig. 1 that the converted MgYNi_4_ was largely absent from the subsequent peritectic reaction and was retained in the Mg_2−x_Y_x_Ni_0.9_Co_0.1_ alloy.

### Microstructures of the as-cast alloys

**Figure 3:**
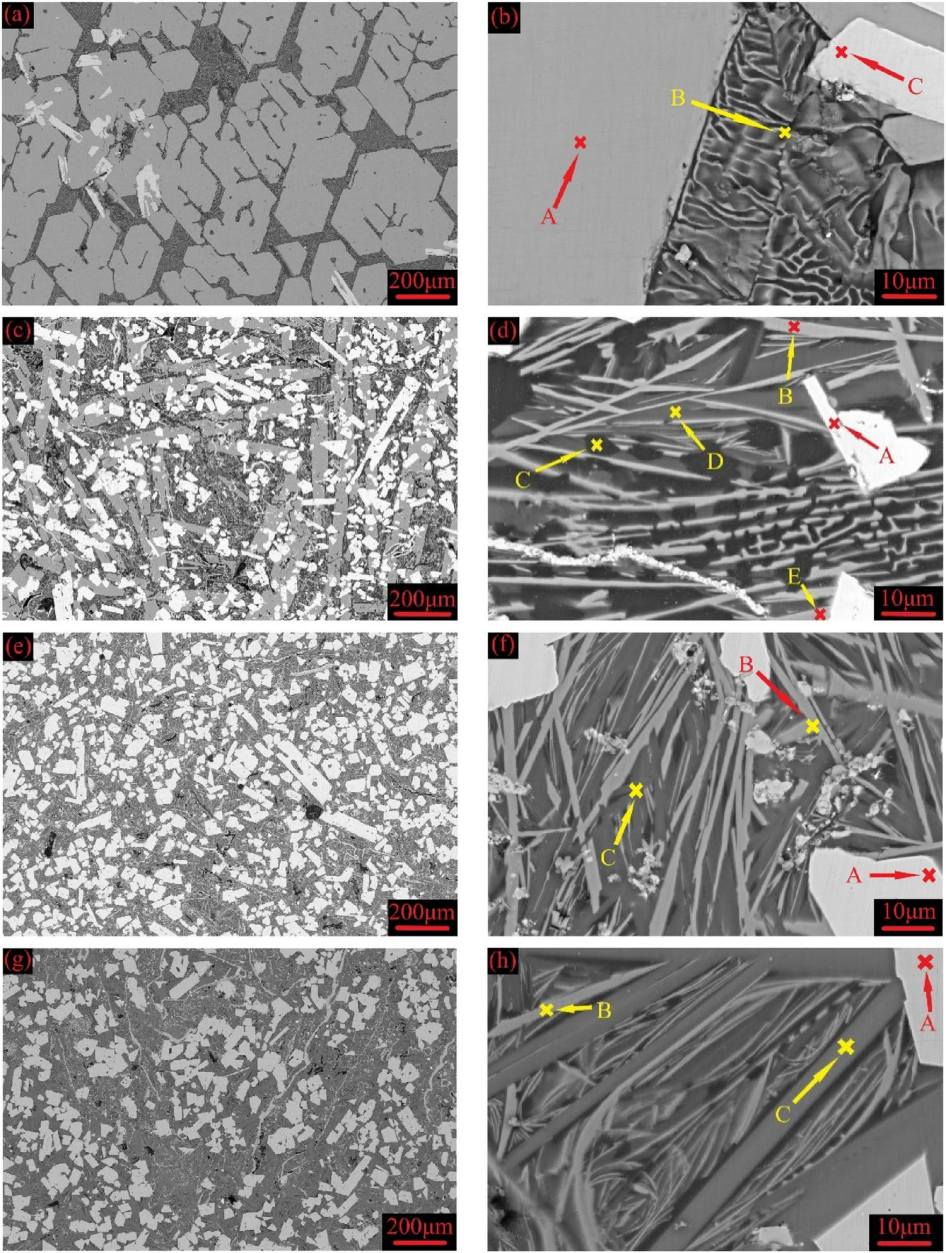
SEM/BSE images of the as-cast Mg_2−x_Y_x_Ni_0.9_Co_0.1_ alloys at low and high magnification for (**a** and **b**) x = 0, (**c** and **d**) x = 0.2, (**e** and **f**) x = 0.3, and (**g** and **h**) x = 0.4.

The scanning electron microscope equipped with backscattered electrons detector (SEM/BSE) images of the as-cast Mg_2−x_Y_x_Ni_0.9_Co_0.1_ (x = 0, 0.2, 0.3, and 0.4) alloys are shown in Fig. 3, and the corresponding EDS results are listed in Table 1. Based on the SEM images, EDS results, and the solidification path discussed, the as-cast Mg_2_Ni_0.9_Co_0.1_ was composed of gray block peritectic Mg_2_Ni and dark lamellar eutectic Mg–Mg_2_Ni, with some bright fine primary MgNi(Co)_2_ dispersed in the matrix, as shown in Fig. 3a and b. Meanwhile, the remaining primary MgNi(Co)_2_ was not found in the XRD test due to its small quantity and fine dispersion distribution characteristics.

**Table 1:** EDS results of the as-cast Mg_2−x_Y_x_Ni_0.9_Co_0.1_ (x = 0, 0.2, 0.3, and 0.4) alloys.

Alloy	Position	Weight%				Atomic%				Phase
		Mg	Y	Ni	Co	Mg	Y	Ni	Co	
Mg_2_Ni_0.9_Co_0.1_	A	48.06	–	50.3	1.91	69.09	–	29.78	1.13	Mg_2_Ni
	B	69.4	–	29.4	1.11	84.5	–	14.8	0.5	Mg + Mg_2_Ni
	C	20.8	–	68.2	10.8	38.9	–	52.7	8.3	MgNi(Co)_2_
Mg_1.8_Y_0.2_Ni_0.9_Co_0.1_	A	8.19	24.6	67.1	–	19.1	15.7	65.0	–	MgYNi_4_
	B	47.5	6.42	45.9	–	69.5	2.57	27.8	–	Mg_2_Ni
	C	100	0	0	–	100	0	0	–	$\alpha$-Mg
	D	79.5	11.8	8.56	–	92.1	3.76	4.11	–	$\varepsilon$-Mg
	E	44.1	4.44	51.4	–	66.2	1.82	31.9	–	Mg_2_Ni
Mg_1.7_Y_0.3_Ni_0.9_Co_0.1_	A	8.0	25.6	66.3	–	18.8	16.5	64.6	–	MgYNi_4_
	B	47.7	7.22	45.0	–	69.8	2.89	27.2	–	Mg_2_Ni
	C	75.1	14.0	10.7	–	90.0	4.6	5.35	–	$\varepsilon$-Mg
Mg_1.6_Y_0.4_Ni_0.9_Co_0.1_	A	8.68	27.0	64.2	–	20.3	17.3	62.3	–	MgYNi_4_
	B	46.3	9.42	44.2	–	68.9	3.83	27.2	–	Mg_2_Ni
	C	68.4	20.2	11.2	–	87.0	7.04	5.94	–	$\varepsilon$-Mg

As shown in Fig. 3c,e, and g, Mg_2−x_Y_x_Ni_0.9_Co_0.1_(x = 0.2, 0.3, and 0.4) alloys had largely increased amounts of the bright phase compared with Mg_2_Ni_0.9_Co_0.1_. Additionally, the corresponding EDS results showed that they were primary MgYNi_4_, coinciding well with the XRD results. Meanwhile, the original peritectic Mg_2_Ni block was refined and elongated after a small Y content (x = 0.2) was added, as shown in Fig. 3c. Furthermore, the primary precipitation phase was all MgYNi_4_, and the subsequent peritectic reaction was inhibited when the Y content increased above 0.3, resulting in the disappearance of peritectic Mg_2_Ni. Consequently, the Mg_1.7_Y_0.3_Ni_0.9_Co_0.1_ and Mg_1.6_Y_0.4_Ni_0.9_Co_0.1_ alloys were only composed of bright primary MgYNi_4_ and dark lamellar eutectic Mg-Mg_2_Ni.

Unlike the Mg_2_Ni_0.9_Co_0.1_ alloy, the Mg_1.8_Y_0.2_Ni_0.9_Co_0.1_ alloy had four regions with contrasting degrees. The results obtained using EDS analysis of the composition of each region are shown in Table 1. Combined with the XRD results of the alloys, it could be seen that the bright white region A embedded in the matrix was the first precipitation of the MgYNi_4_ phase. The elongated acicular gray region B was the eutectic Mg_2_Ni phase, the black region C around the acicular Mg_2_Ni phase was the eutectic α-Mg phase (Mg phase without a solid solution of other elements), and the gray-black region D was the eutectic ε-Mg phase (Mg phase with small amounts of Ni and Y elements). The elongated gray region E was the matrix phase of peritectic Mg_2_Ni. As seen in Fig. 3c, the region composed of B, C, and D was located in the gap of the peritectic Mg_2_Ni matrix phase, and it should be the eutectic structure composed of Mg and Mg_2_Ni. Compared with the Mg_2_Ni_0.9_Co_0.1_ alloy, the eutectic structure of the Mg_1.8_Y_0.2_Ni_0.9_Co_0.1_ alloy was finer and not lamellar. Simultaneously, the peritectic Mg_2_Ni phase changed from a thick block to a long strip, indicating that the addition of Y benefitted in refining the microstructure of the alloy.

Compared with the Mg_1.8_Y_0.2_Ni_0.9_Co_0.1_ alloy, the peritectic Mg_2_Ni and eutectic α-Mg phases disappeared in the Mg_1.7_Y_0.3_Ni_0.9_Co_0.1_ alloy. In other words, the first precipitated MgNi_2_ phase of the alloy was converted into MgYNi_4_ under the condition of non-equilibrium solidification when the added Y content increased to 0.3, but MgYNi_4_ did not participate in the peritectic reaction, leading to the precipitation of no peritectic Mg_2_Ni phase in the alloy. With the further decrease in temperature, the eutectic reaction occurred in the remaining alloy melt, forming the eutectic structure. Due to the high Y content in the melt, Y was dissolved in α-Mg in the eutectic structure to convert it to $\varepsilon$-Mg. Furthermore, the EDS analysis showed that the phase composition of the alloy did not change when the added Y content was increased to 0.4. Mg_1.6_Y_0.4_Ni_0.9_Co_0.1_ and Mg_1.7_Y_0.3_Ni_0.9_Co_0.1_ had the same solidification path, where MgYNi_4_ was precipitated first, followed by the precipitation of Mg_2_Ni and ε-Mg through eutectic reactions.

**Figure 4:**
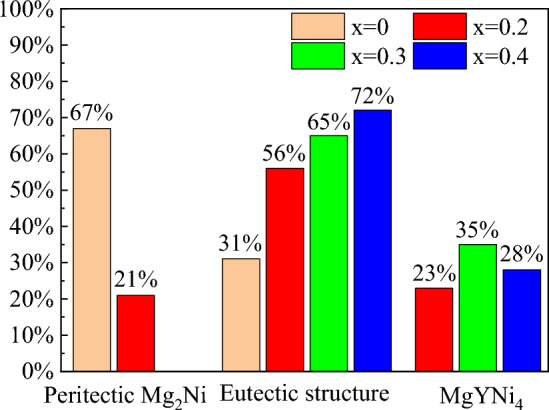
Phase area ratios in Mg_2−x_Y_x_Ni_0.9_Co_0.1_ (x = 0, 0.2, 0.3, and 0.4) alloys.

The phase area ratios in the SEM images were counted using IPP to describe the effect of Y addition on the microstructure of the alloy more intuitively, and the corresponding statistically calculated results are shown in Fig. 4. Clearly, the peritectic reaction was inhibited when the added Y content was above 0.2. Compared with Mg_2_Ni_0.9_Co_0.1_, the area ratio of the peritectic Mg_2_Ni decreased from 67 to 21% when a small amount of Y (x = 0.2) was added. Meanwhile, more melt was retained at low temperatures to conduct the eutectic reaction since the peritectic reaction was inhibited, increasing the area ratio of eutectic Mg–Mg_2_Ni with increased Y content. The maximum area ratio for MgYNi_4_ was obtained when the added Y content was 0.3. Thus, the added Y was first dissolved in primary MgNi_2_, which was converted into MgYNi_4_ when Y reached the corresponding content in the Mg_2−x_Y_x_Ni_0.9_Co_0.1_ alloy. The solidification path was L → L + Mg(Y)Ni_2_ → L + MgYNi_4_ + Mg(Y)Ni_2_ → L + MgYNi_4_ + Mg_2_Ni(peritectic) → MgYNi_4_ + Mg_2_Ni(peritectic) + Mg–Mg_2_Ni(eutectic). Notably, all Mg(Y)Ni_2_ would be converted into MgYNi4 when the added Y content was above 0.2, inhibiting the peritectic reaction and disappearance of peritectic Mg_2_Ni in the final solidified structures. Thus, the MgYNi_4_ amount in the final solidified microstructure depended on the amounts of primary Mg(Y)Ni2 and Y that could participate in the reaction during solidification. The amount of the primary Mg(Y)Ni_2_ decreased when the added Y content was further increased from 0.3 to 0.4, reducing the final transformed MgYNi_4_, as shown in Fig. 4. The excess Y will dissolved into Mg–Mg_2_Ni eutectic during subsequent solidification, resulting in the higher Y content dissolved in eutectic Mg-Mg_2_Ni in Mg_1.6_Y_0.4_Ni_0.9_Co_0.1_ than that in Mg_1.7_Y_0.3_Ni_0.9_Co_0.1_ and Mg_1.8_Y_0.2_Ni_0.9_Co_0.1_, as shown in Table 1. In addition to the phase composition discussed, adding Y significantly refined the solidification structure, as seen in Fig. 3.

### Activation and de-/hydrogenation properties

Since an impurity layer composed of oxides and hydroxides is usually formed on the surface of ball-milled alloy particles, activation is necessary to break this surface impurity layer and expose the fresh alloy metal inside. This study carried out the activation through three successive de-/hydrogenation circles at 300 °C. Hydrogenation and dehydrogenation were conducted under 2.5 and 0.1 MPa hydrogen pressure, respectively. Meanwhile, this study tested and discussed only the activation and de-/hydrogenation properties of Mg_2−x_Y_x_Ni_0.9_Co_0.1_ (x = 0, 0.2, and 0.4) since the peritectic reaction was inhibited and Mg_1.7_Y_0.3_Ni_0.9_Co_0.1_ exhibited microstructures similar to that of Mg_1.6_Y_0.4_Ni_0.9_Co_0.1_.

**Figure 5:**
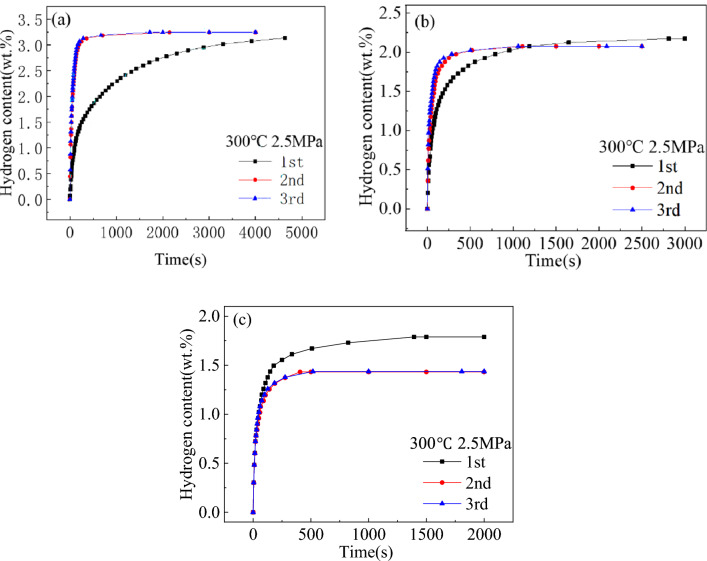
Activation hydrogen absorption curves of Mg_2−x_Y_x_Ni_0.9_Co_0.1_ alloys for (**a**) x = 0, (**b**) x = 0.2, and (**c**) x = 0.4.

The activation hydrogen absorption curves of the Mg_2−x_Y_x_Ni_0.9_Co_0.1_ (x = 0, 0.2, and 0.4) alloys are shown in Fig. 5. Comparing the time required to reach the maximum hydrogen absorption capacity for the first hydrogen absorption curve, the hydrogen absorption rate of the three alloys in descending order was: Mg_1.6_Y_0.4_Ni_0.9_Co_0.1_, Mg_1.8_Y_0.4_Ni_0.9_Co_0.1_, and Mg_2_Ni_0.9_Co_0.1_. Unlike the hydrogen absorption rate, the maximum hydrogen absorption capacity in descending order was: Mg_2_Ni_0.9_Co_0.1_ (3.13 wt%), Mg_1.8_Y_0.4_Ni_0.9_Co_0.1_ (2.17 wt%), and Mg_1.6_Y_0.4_Ni_0.9_Co_0.1_ (1.79 wt%). Therefore, the addition of Y improved the hydrogen absorption rate but decreased the hydrogenation capacity. Additionally, the third hydrogen absorption curve coincided with the second hydrogen absorption curve, indicating that the three alloys could be completely activated after two successive de-/hydrogenation circles.

Unlike Mg_2_Ni_0.9_Co_0.1_, Mg_1.8_Y_0.2_Ni_0.9_Co_0.1_ and Mg_1.6_Y_0.4_Ni_0.9_Co_0.1_ had slightly lower hydrogen absorption capacities after activation than before activation due to the formation of YH_3_. When Y is added, YH_3_ is formed during hydrogen absorption, followed by conversion to YH_2_ during dehydrogenation. The dehydrogenation temperature of YH_2_ is about 1063 K^27^, which is obviously much higher than the activation temperature in this study. Consequently, YH_2_ could not be dehydrogenated and was left, resulting in the decreased hydrogen absorption capacity of the alloy in the subsequent hydrogen absorption process. Meanwhile, Mg_1.6_Y_0.4_Ni_0.9_Co_0.1_ had a more obvious reduction in the hydrogen absorption capacity after activation since Mg_1.6_Y_0.4_Ni_0.9_Co_0.1_ had a larger Y content than Mg_1.8_Y_0.2_Ni_0.9_Co_0.1_, as shown in Fig. 5b and c.

Due to its low average atomic density and the large space between atoms, hydrogen diffused more easily through the phase boundary^28^. As discussed in Section “Microstructures of the as-cast alloys”, the area ratio of eutectic structures increased, and the solidification structure was refined with the increased Y content, improving the activation property of the alloys.

**Figure 6:**
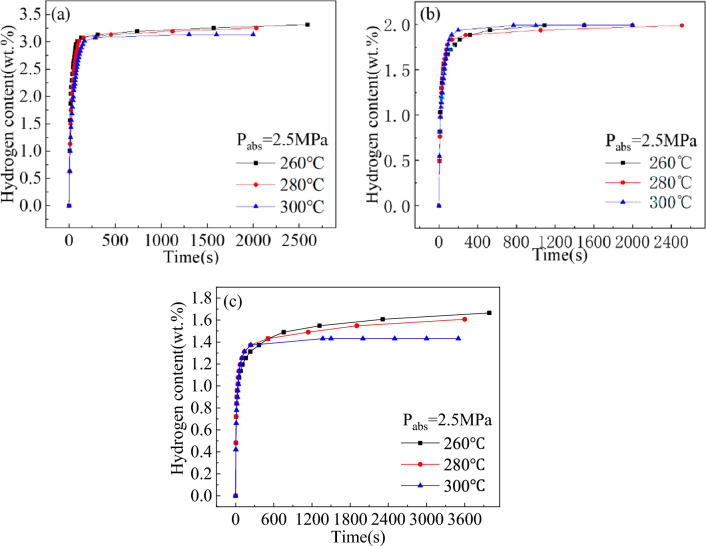
Hydrogen absorption kinetic curves of Mg_2−x_Y_x_Ni_0.9_Co_0.1_ alloys at different temperatures for (**a**) x = 0, (**b**) x = 0.2, and (**c**) x = 0.4.

The hydrogen absorption capacity and rate at different temperatures are important indexes to reflect the hydrogen absorption kinetics of the alloys. The hydrogen absorption kinetic curves of Mg_2−x_Y_x_Ni_0.9_Co_0.1_ (x = 0, 0.2, and 0.4) alloys at different temperatures are shown in Fig. 6. All the three alloys showed a fast hydrogen absorption rate after being fully activated, reaching more than 80% of the corresponding maximum hydrogen absorption capacity in only 6 min. As shown in Section “Microstructures of the as-cast alloys”, MgYNi_4_ was formed when Y was added, and its proportion increased with the increased Y content. The maximum hydrogen absorption capacity of Mg_2−x_Y_x_Ni_0.9_Co_0.1_ (x = 0, 0.2, and 0.4) decreased with the increased Y content, from 3.31 wt% to 1.99 wt% and then to 1.67 wt% at 260 °C due to the presence of the non-hydrogen absorbing phase.

The maximum hydrogen absorption capacity of the Mg_2_Ni_0.9_Co_0.1_ alloy was 3.13 wt% at 2.5 MPa hydrogen pressure and 300 °C temperature, and the time taken to reach the maximum hydrogen absorption capacity was 22 min. Under the same reaction conditions, Mg_2−x_Y_x_Ni_0.9_Co_0.1_ (x = 0.2 and 0.4) alloys could reach more than 90% of the maximum hydrogen absorption capacity in 200 s. By comparison, the maximum hydrogen absorption of the alloys decreased with the addition of Y due to the formation of the MgYNi_4_ phase that does not absorb hydrogen and the unsaturated hydride Mg_2_NiH_0.3_ in the Mg_2−x_Y_x_Ni_0.9_Co_0.1_ (x = 0.2 and 0.4) alloys during the hydrogen absorption process.

**Figure 7:**
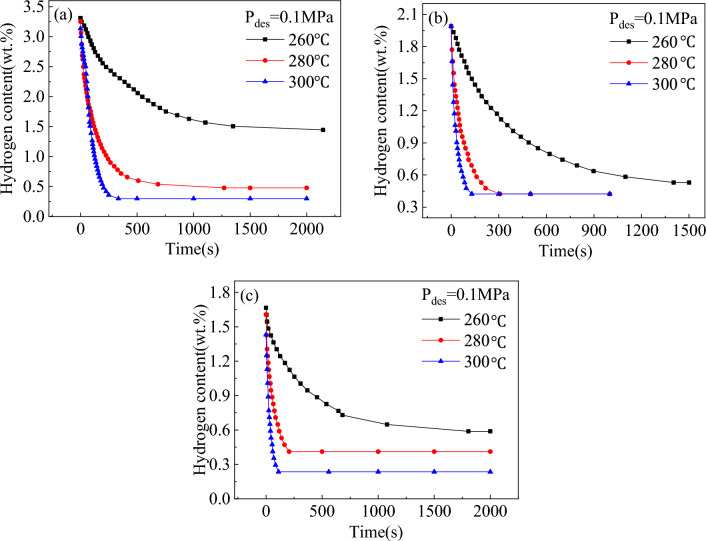
Hydrogen desorption kinetic curves of the Mg_2−x_Y_x_Ni_0.9_Co_0.1_ alloys at different temperatures for (**a**) x = 0, (**b**) x = 0.2, and (**c**) x = 0.4.

The hydrogen desorption kinetic curves of Mg_2−x_Y_x_Ni_0.9_Co_0.1_ (x = 0, 0.2, and 0.4) alloys at 0.1 MPa hydrogen pressure after hydrogen absorption under the conditions mentioned are shown in Fig. 7. The comparison of the de-/hydrogenation kinetic curves of the alloys showed that the complete hydrogen desorption of alloys required significantly lower time than the hydrogen absorption process. The hydride layer on the surface of the alloys hinders the diffusion of hydrogen atoms into the alloys during their hydrogen absorption process. However, this hydride layer on the surface of the alloys breaks down during the desorption process to produce hydrogen atoms that do not need to pass through the hydride layer but only through the metal surface. Since the diffusion rate of the hydrogen atoms in the hydride was much lower than that in the metal surface^29^, the complete hydrogen desorption of the alloys required significantly lower time than the complete hydrogen absorption. Additionally, the maximum hydrogen discharges of the three alloys increased with the increase in temperature since the dehydrogenation of the alloy is a reversible process and the hydrogen absorption and desorption reactions occur simultaneously, where the absorption and desorption processes are exothermic and endothermic reactions, respectively. The hydrogen desorption of the alloys would increase with the increase in temperature since the temperature is conducive to the desorption process. The dehydrogenation capacities of the Mg_2−x_Y_x_Ni_0.9_Co_0.1_ (x = 0, 0.2, and 0.4) alloys at different temperatures for 50 s are shown in Table 2. The dehydrogenation amounts of the alloys for x = 0, 0.2, and 0.4 were 0.19, 0.22, and 0.24 wt% after hydrogen desorption for 50 s at 260 °C, respectively, indicating that the addition of Y improved the desorption kinetic properties of the alloys. However, this phenomenon was not observed at higher reaction temperatures.

**Table 2:** Dehydrogenation capacities of the Mg_2−x_Y_x_Ni_0.9_Co_0.1_ (x = 0, 0.2, and 0.4) alloys at different temperatures.

Alloy	Dehydrogenation Capacity (wt%)		
	260 °C	280 °C	300 °C
x = 0	0.19	1.13	0.76
x = 0.2	0.22	0.87	1.25
x = 0.4	0.24	0.66	0.9

### Hydrogen absorption reaction mechanism of alloys

**Figure 8:**
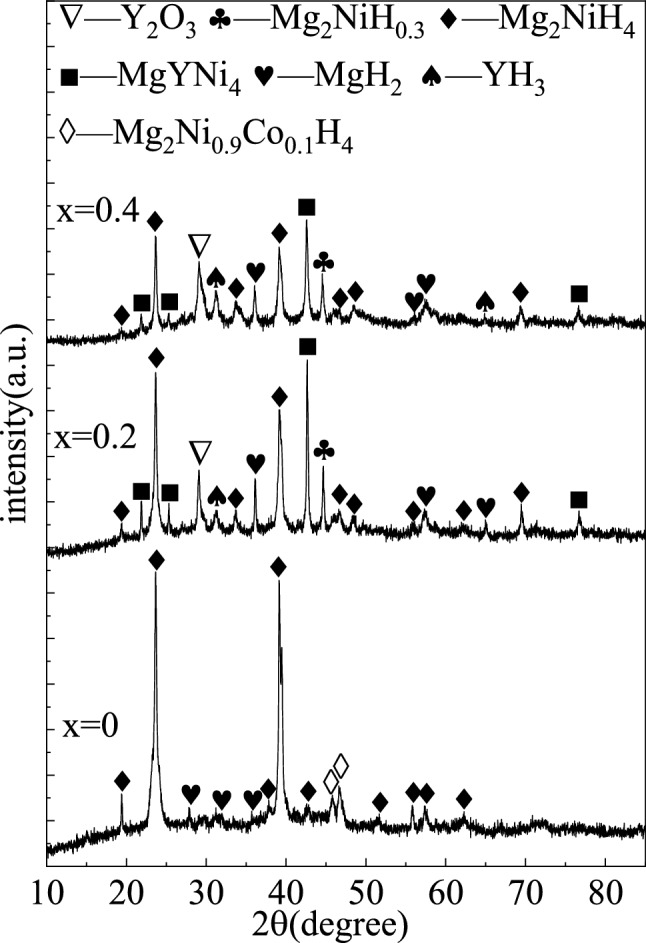
XRD patterns of the Mg_2−x_Y_x_Ni_0.9_Co_0.1_ (x = 0, 0.2, 0.4) alloy hydrides.

The XRD patterns of the Mg_2−x_Y_x_Ni_0.9_Co_0.1_ (x = 0, 0.2, and 0.4) alloy hydrides after hydrogen absorption at 300 °C and 2.5 MPa are shown in Fig. 8. The Mg_2_Ni_0.9_Co_0.1_ alloy hydride was mainly composed of Mg_2_NiH_4_, MgH_2_, and Mg_2_Ni_0.9_Co_0.1_H_4_. The Mg_2_(Ni, Co) solid solution formed in Mg_2_Ni by partial Co solution reacted with hydrogen to form Mg_2_Ni_0.9_Co_0.1_H_4_. According to Hayakawa et al. Mg_2_Ni_0.9_Co_0.1_H_4_ is another polymorph of Mg_2_NiH_4_, the distortion product of the cubic HT- Mg_2_NiH_4_^30^.

Combined with the XRD patterns and EDS analysis, the hydrogen absorption reaction of the Mg_2_Ni_0.9_Co_0.1_ alloy can be expressed as follows:

1 $$ {\text{Mg}} + {\text{H}}_{{2}} \to {\text{MgH}}_{{2}} $$

2 $$ {\text{Mg}}_{{2}} {\text{Ni}} + {\text{2H}}_{{2}} \to {\text{Mg}}_{{2}} {\text{NiH}}_{{4}} $$

3 $$ {\text{Mg}}_{{2}} \left( {{\text{Ni}},{\text{ Co}}} \right) + {\text{2H}}_{{2}} \to {\text{Mg}}_{{2}} {\text{Ni}}_{{0.{9}}} {\text{Co}}_{{0.{1}}} {\text{H}}_{{4}} $$

Additionally, no other Co phases except the Mg_2_Ni_0.9_Co_0.1_H_4_ phase were found in the XRD pattern of the Mg_2_Ni_0.9_Co_0.1_ alloy hydride. Therefore, it could be concluded that the Mg–Ni–Co phase did not undergo hydrogen absorption or decomposition reaction under the experimental conditions of this study, which is consistent with the fact that MgNi_2_ in the Mg_2_Ni alloy did not undergo the hydrogen absorption reaction^31^.

The XRD patterns of the Mg_2−x_Y_x_Ni_0.9_Co_0.1_ (x = 0.2 and 0.4) alloys after hydrogen absorption showed that both of them were composed of Mg_2_NiH_4_, MgH_2_, MgYNi_4_, YH_3_, Y_2_O_3_, and Mg_2_NiH_0.3_. The Mg_2_NiH_0.3_ phase was an intermediate product in the hydrogen absorption reaction of Mg_2_Ni to form Mg_2_NiH_4_, an unsaturated hydride. Since the samples were ball-milled in the argon atmosphere and the operation of taking out the samples and loading them into the reactor was performed in a glove box, the trace Y element in the alloy would not be completely oxidized, and YH_3_ would be formed in the hydrogen absorption process. The intensity of the YH_3_ diffraction peak increased slightly with the increased Y content, indicating that more Y was involved in the reaction to form YH_3_ in the hydrogen absorption process of the alloys with the increased substitution of Y.

Combined with the XRD patterns and EDS analysis, the hydrogen absorption reaction of the Mg_2−x_Y_x_Ni_0.9_Co_0.1_ (x = 0.2 and 0.4) alloys can be expressed as follows:

4 $$ {\text{Mg}} + {\text{H}}_{{2}} \to {\text{MgH}}_{{2}} $$

5 $$ {\text{Mg}}_{{2}} {\text{Ni}} + {\text{H}}_{{2}} \to {\text{Mg}}_{{2}} {\text{NiH}}_{{0.{3}}} $$

6 $$ {\text{Mg}}_{{2}} {\text{NiH}}_{{0.{3}}} + {\text{H}}_{{2}} \to {\text{Mg}}_{{2}} {\text{NiH}}_{{4}} $$

7 $$ {\text{Y}} + {\text{H}}_{{2}} \to {\text{YH}}_{{3}} $$

### Hydrogen release process of alloys hydride

**Figure 9:**
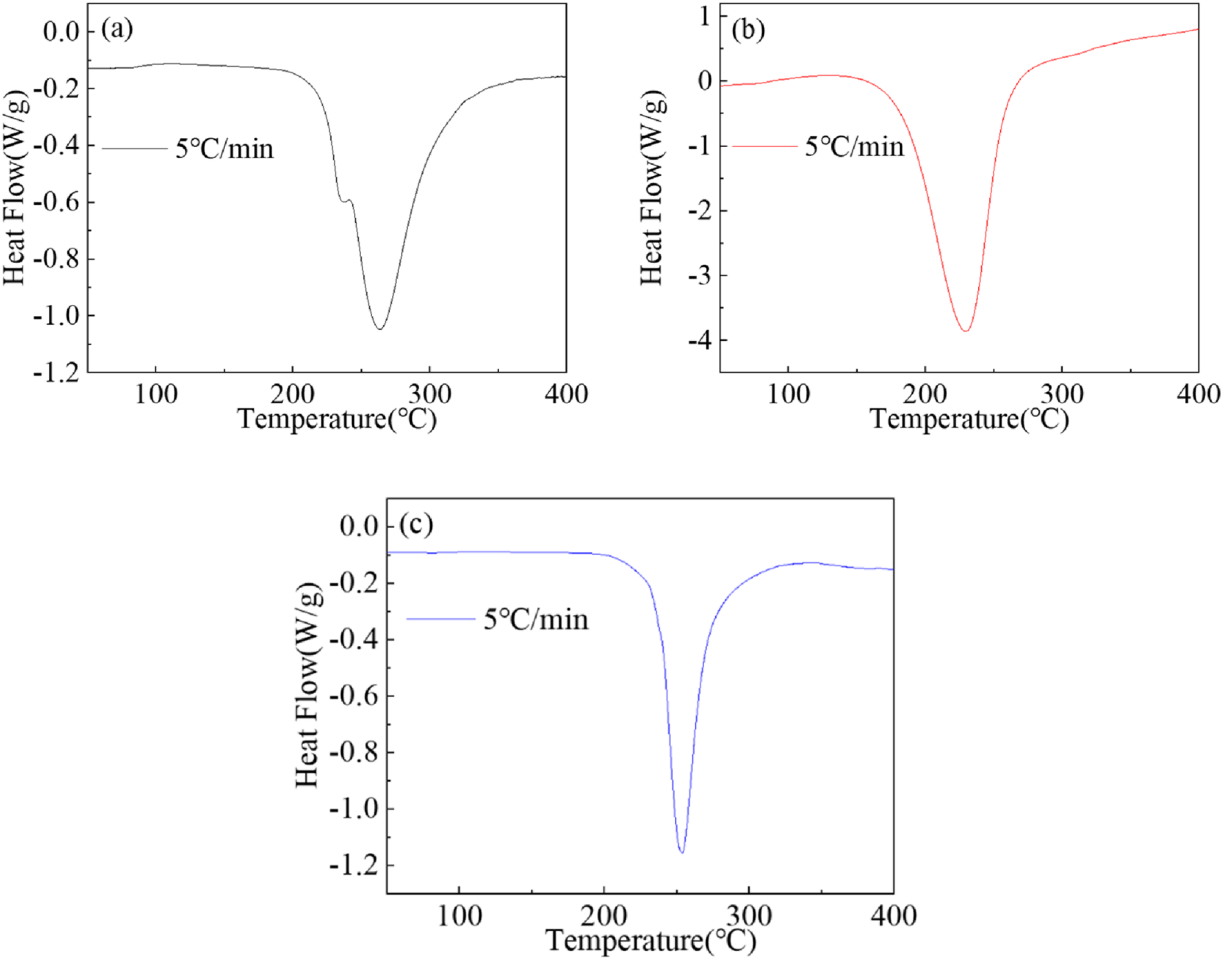
DSC curves of the dehydrogenation of the Mg_2−x_Y_x_Ni_0.9_Co_0.1_ alloy hydrides for (**a**) x = 0, (**b**) x = 0.2, and (**c**) x = 0.4.

The phase transition of the Mg_2−x_Y_x_Ni_0.9_Co_0.1_ (x = 0, 0.2, and 0.4) alloy hydrides during dehydrogenation was studied using DSC measurements. The DSC curves of the Mg_2−x_Y_x_Ni_0.9_Co_0.1_ (x = 0, 0.2, and 0.4) alloy hydrides at a heating rate of 5 °C/min are shown in Fig. 9. The DSC curve of the Mg_2_Ni_0.9_Co_0.1_ alloy hydride had two endothermic peaks, with the smaller endothermic peak at 236 °C. The main endothermic peak had a peak temperature of 263.5 °C. The XRD pattern of the Mg_2_Ni_0.9_Co_0.1_ alloy hydride showed that the hydrogen desorption phases included Mg_2_NiH_4_, MgH_2_, and Mg_2_Ni_0.9_Co_0.1_H_4_. Mg_2_Ni_0.9_Co_0.1_H_4_ was a metastable phase produced by the reaction between the Mg_2_(Ni, Co) solid solution and hydrogen. As reported in the literature, the peak desorption temperatures of Mg_2_NiH_4_ and MgH_2_ were 247–287 °C and 327–387 °C, respectively^32^. It was inferred that the endothermic peak at 236 °C corresponded to the dehydrogenation process of the metastable Mg_2_Ni_0.9_Co_0.1_H_4_, and the main endothermic peak at 263.5 °C corresponded to the hydrogen desorption process of the Mg_2_NiH_4_ and MgH_2_ phases. Thus, the main endothermic peak was the superposition of the Mg_2_NiH_4_ and MgH_2_ dehydrogenation peaks.

The DSC curves of the Mg_2−x_Y_x_Ni_0.9_Co_0.1_ (x = 0.2 and 0.4) alloy hydrides had only one endothermic peak after adding Y. The corresponding peak temperatures for x = 0.2 and 0.4 were 230 and 254 °C, respectively. The XRD analysis of the Mg_2−x_Y_x_Ni_0.9_Co_0.1_ (x = 0.2 and 0.4) alloy hydrides showed that Mg_2_NiH_4_, MgH_2_, YH_3_, and Mg_2_NiH_0.3_ were the four kinds of decomposable hydrides after hydrogen absorption. Based on DSC curves and XRD analysis, the four hydrides of the desorption processes of the Mg_2−x_Y_x_Ni_0.9_Co_0.1_ (x = 0.2 and 0.4) alloy completely overlapped due to the synergistic effect in the process of hydride desorption.

Comparing the DSC curves of the three alloys, it could be easily determined that the initial dehydrogenation temperature of Mg_2_Ni_0.9_Co_0.1_ alloy was about 200 °C, and the dehydrogenation temperature of Mg_2_NiH_4_ was 253 °C^33^. This indicates that adding Co could reduce the stability of the alloy hydride, reducing the corresponding hydrogen desorption temperature. The initial desorption temperatures of the Mg_1.8_Y_0.2_Ni_0.9_Co_0.1_ and Mg_1.6_Y_0.4_Ni_0.9_Co_0.1_ alloys were 156 and 208 °C, respectively, indicating that Y was also an effective instability agent, and its addition could reduce the desorption temperatures of the alloys. However, the added Y content affected the improvement degree. The initial hydrogen desorption temperature decreased and then increased with the increased Y content. The improvement was the best when x = 0.2.

**Figure 10:**
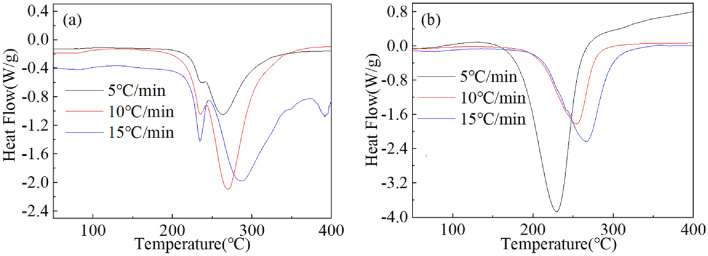
DSC curves of the dehydrogenation of the Mg_2−x_Y_x_Ni_0.9_Co_0.1_ alloy hydrides at different heating rates for (**a**) x = 0 and (**b**) x = 0.2.

The DSC curves of the Mg_2−x_Y_x_Ni_0.9_Co_0.1_ (x = 0 and 0.2) alloy hydrides at different heating rates of 5, 10, and 15 °C/min are shown in Fig. 10. The peak desorption temperatures of the alloys at different heating rates are presented in Table 3. The heating rate affected the peak desorption temperature of the alloy hydrides. The peak desorption temperature increased for both alloys with the increase in the heating rate. The dehydrogenation activation energy ${\text{E}}_{\text{a}}$ is the height of the barrier between the lowest potential energy of the dehydrogenation reactant and the product. The kinetic properties of hydrogen desorption were better for lower activation energies. Furthermore, this study used the peak temperatures of the DSC curves with different heating rates to investigate the dehydrogenation kinetics of the Mg_2−x_Y_x_Ni_0.9_Co_0.1_ (x = 0 and 0.2) alloys. The dehydrogenation activation energy of the Mg_2−x_Y_x_Ni_0.9_Co_0.1_ (x = 0 and 0.2) alloy was calculated according to the Kissinger equation shown in Eq. 8.

8 $$ \ln \frac{\beta }{{T_{P}^{2} }} = A - \frac{{E_{a} }}{{RT_{P} }} $$

where, $\beta$, $T_{P}$, ${\text{E}}_{\text{a}}$, *R*, and *A* represent the linear heating rate (°C/min or K/min), the dehydrogenation peak temperature (K), the activation energy for hydrogen desorption (kJ/mol), the gas constant (8.314 J/mol/K), and a linear constant, respectively.

**Table 3:** Dehydrogenation peak temperatures of the Mg_2−x_Y_x_Ni_0.9_Co_0.1_ (x = 0 and 0.2) alloy hydrides at heating rates of 5, 10, and 15 °C/min.

Alloy	Peak desorption temperature (°C)		
	5 °C/min	10 °C/min	15 °C/min
x = 0	263	270	288
x = 0.2	230	255	268

**Figure 11:**
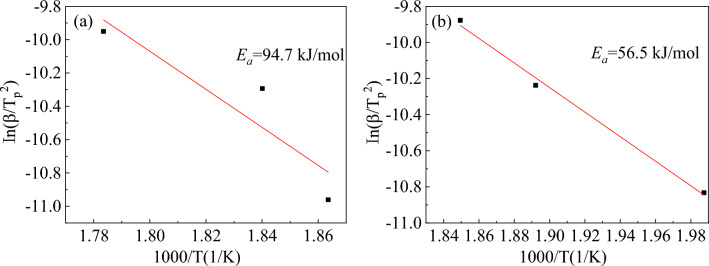
The fitted Kissinger curves of the Mg_2−x_Y_x_Ni_0.9_Co_0.1_ alloy hydrides for (**a**) x = 0 and (**b**) x = 0.2.

As shown in Fig. 11, the slope of the plot of ${\text{ln}}\left( {\beta {\text{/T}}_{{\text{P}}}^{{2}} } \right)$ versus 1/$T_{P}$ was ${\text{E}}_{\text{a}}$/R. Using the slope of the straight line, the calculated dehydrogenation activation energy of the Mg_2_Ni_0.9_Co_0.1_ alloy was 94.7 kJ/mol, slightly lower than the dehydrogenation activation energy of Mg_2_NiH_4_ (102 kJ/mol)^34^. The dehydrogenation activation energy of the Mg_1.8_Y_0.2_Ni_0.9_Co_0.1_ alloy was 56.5 kJ/mol, which was much lower than that of the Mg_2_Ni_0.9_Co_0.1_ alloy. The experimental results showed that adding Co and Y could reduce the activation energy of the hydrogen desorption reaction of the Mg_2_Ni alloy and improve its hydrogen desorption kinetics. Additionally, the synergistic effect of Co and Y was more obvious than the single addition of Co, which is consistent with the analysis of the hydrogen desorption kinetic curves. The improvement of the dehydrogenation kinetics could be explained from two aspects. The eutectic structure of the alloy was increased and refined with the increase in the added Y content. Several eutectic phase boundaries in the eutectic structure provided an effective way to diffuse hydrogen atoms in the alloy matrix. YH_3_ in the hydride could promote the decomposition of MgH_2_ in the desorption process, speeding up the desorption reaction rate of the alloys^18^.

## Conclusions

This study prepared Mg_2−x_Y_x_Ni_0.9_Co_0.1_ (x = 0, 0.2, 0.3, and 0.4) alloys using a metallurgical method to investigate their microstructures and hydrogen storage properties and discuss the effects of the addition of Y on the microstructure and hydrogen storage properties of these alloys. The main conclusions are as follows:

1. The as-cast Mg_2_Ni_0.9_Co_0.1_ alloy was composed of the peritectic Mg_2_Ni, eutectic Mg–Mg_2_Ni, and a small amount of the first precipitated Mg–Ni–Co ternary phase. In the absorption process, the Mg_2_Ni phase was converted to the Mg_2_NiH_4_ phase, the Mg_2_(Ni, Co) solid solution reacted with hydrogen to form Mg_2_Ni_0.9_Co_0.1_H_4_, and the Mg phase was converted to the MgH_2_ phase. The Mg–Ni–Co ternary phase was precipitated without hydrogen absorption. After the addition of Y, the MgYNi_4_ phase and the trace Y_2_O_3_ phase, along with the Mg and Mg_2_Ni phases, were observed in the XRD patterns of the Mg_2−x_Y_x_Ni_0.9_Co_0.1_ (x = 0.2, 0.3, and 0.4) alloys. The phase of the alloy after hydrogen absorption was composed of Mg_2_NiH_4_, MgH_2_, MgYNi_4_, YH_3_, Y_2_O_3_, and Mg_2_NiH_0.3_.
2. The area ratio of eutectic Mg–Mg_2_Ni in the Mg_2−x_Y_x_Ni_0.9_Co_0.1_ (x = 0, 0.2, 0.3, and 0.4) alloys increased, and the eutectic structure was refined with the increased Y content. However, the area ratio of the peritectic Mg_2_Ni phase gradually decreased until it disappeared with the increased Y content. In addition to the first precipitated MgYNi_4_, Y could be dissolved in Mg and Mg_2_Ni, increasing the lattice parameters of the corresponding phase.
3. The test and analysis of the activation properties and de-/hydrogenation kinetics of the alloys showed that the addition of Y improved the activation properties of the alloys. The alloy only needed one cycle to complete the activation when x = 0.4. Although the addition of Y would improve the hydrogen desorption kinetics, it would decrease the hydrogen storage capacity of the alloys.
4. The addition of Y and Co could decrease the initial dehydrogenation temperature of the Mg_2_Ni alloy, and their synergistic effect was more significant than the addition of Co. Combined with hydrogen storage capacity, activation performance, hydrogen desorption temperature, and kinetics, Mg_1.8_Y_0.2_Ni_0.9_Co_0.1_ had the best comprehensive hydrogen storage performance. The maximum hydrogen absorption capacity, the initial desorption temperature, and the dehydrogenation activation energy were 2.07 wt%, 156 °C, and 56.5 kJ/mol, respectively, after complete activation.

## Data Availability

All data generated or analysed during this study are included in this published article.
